# The Neoplastic Side of the Abdominal Wall: A Comprehensive Pictorial Essay of Benign and Malignant Neoplasms

**DOI:** 10.3390/diagnostics13020315

**Published:** 2023-01-15

**Authors:** Giorgia Porrello, Roberto Cannella, Eduardo Alvarez-Hornia Pérez, Giuseppe Brancatelli, Federica Vernuccio

**Affiliations:** 1 Section of Radiology, Department of Biomedicine, Neuroscience and Advanced Diagnosis (Bi.N.D), University of Palermo, Via del Vespro 129, 90127 Palermo, Italy; 2 Radiology Unit, Department of Diagnostic and Therapeutic Services, IRCCS ISMETT (Mediterranean Institute for Transplantation and Advanced Specialized Therapies), Via Tricomi 5, 90127 Palermo, Italy; 3 Department of Health Promotion, Mother and Child Care, Internal Medicine and Medical Specialties (PROMISE), University of Palermo, Piazza delle Cliniche, 2, 90127 Palermo, Italy; 4 Clinica IMQ Zorrotzaurre, 48014 Bilbao, Spain; 5 Institute of Radiology, Department of Medicine-DIMED, University of Padova, 35128 Padova, Italy

**Keywords:** abdominal wall, incidentaloma, neoplasm, sarcoma, lymphoma, computed tomography, magnetic resonance imaging

## Abstract

Abdominal wall neoplasms are usually benign and, in the majority of these cases, no further work-up or treatment is indicated. The percentage of malignant abdominal neoplasms, however, is not negligible. Radiologists play a pivotal role in identifying imaging features that should favor malignancy, including larger lesion size, edema, neurovascular involvement, and peripheral or inhomogeneous dynamic enhancement, thus indicating to the clinician the need for further work-up. Histopathology is the reference standard for the characterization of abdominal wall neoplasms. In patients undergoing surgery, radiological assessment is needed to guide the surgeon by providing a comprehensive anatomic guide of the tumor extension. We present a pictorial review of benign and malignant abdominal wall neoplasms that can be encountered on radiological examinations, with a main focus on CT and MRI features that help in narrowing the differential diagnosis.

## 1. Introduction

Abdominal wall neoplasms may be encountered incidentally on imaging or may present as palpable masses, leading the patient to the radiologist. Malignancies could be identified in about 40% of cases, based on a study by Bashir et al. [1]. However, this percentage is likely overestimated, as this study was based on a population of 365 patients referred to a sarcoma clinic; therefore, the percentage in the overall population is very likely to be much lower. Hence, in most cases, abdominal wall neoplasms have a benign nature and do not require further work-up or treatment.

Radiologists play a pivotal role in recognizing imaging features that deserve close attention. Imaging features that should favor malignancy in soft-tissue tumors of the abdominal wall include larger lesion size, edema, neurovascular involvement, and peripheral or inhomogeneous dynamic enhancement [2,3].

Ultrasonography (US) is the first-line imaging technique for abdominal wall masses, and it is indicated in patients with palpable masses in the abdominal wall. US plays a main role both for diagnosis and as a guide for interventional procedures and biopsies. In many cases, however, abdominal wall neoplasms are identified incidentally on computed tomography (CT) and magnetic resonance (MR) [1].

CT provides information on tumor extension and involvement of deeper abdominal wall structures, but it is burdened by ionizing radiation exposure. Contrast-enhanced MR is a second-line imaging technique that allows a better understanding of the composition and nature of a mass [2,3]. Histopathology, however, still remains the reference standard for final diagnosis.

Based on the European Society of Musculoskeletal Radiology (ESSR) guidelines on soft tissue tumors, masses above 5 cm should always undergo biopsy after imaging [3].

Benign neoplasms are mainly derived from mesenchymal tissues, with 70% of them classified in six main diagnostic categories: lipomas, fibrous histiocytoma, nodular fasciitis, hemangioma, fibromatosis, and nerve sheath tumors [4,5].

Malignant lesions usually show a larger volume, heterogeneous structure (mainly on MR), and heterogeneous and early contrast enhancement [5]. Around 80% of all abdominal wall malignant primary tumors fall into seven categories: myxofibrosarcoma, liposarcomas, leiomyosarcomas, malignant nerve sheath tumors, synovial cell sarcoma, fibrosarcoma, and sarcoma not otherwise specified (NOS) [4].

This pictorial review aims to provide an overview of the broad spectrum of all these benign and malignant neoplasms, with tips for the differential diagnosis. For each condition, key imaging aspects will be highlighted, in order to provide a complete visual diagnostic guide for the radiologist.

## 2. Imaging Findings: Benign Neoplasms

Table 1 summarizes the main imaging features of benign neoplasms of the abdominal wall.

### 2.1. Lipoma

**Figure 1:**
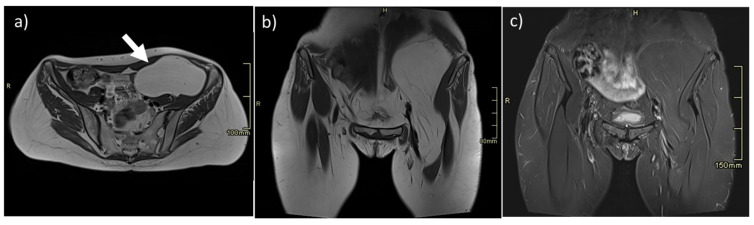
Intramuscular lipoma of the left iliopsoas muscle (arrow). On MR, lipomas are easily studied, as they show well-defined margins and homogeneous fat intensity, as seen on axial (**a**) and coronal (**b**) T2w sequences and homogeneous loss of signal on T2 fat-saturated sequences (**c**).

Lipoma is the most frequent benign soft tissue tumor, with a prevalence ranging from 16% to 50% [1,5,6]. Simple lipomas are homogeneous fatty masses with minimal-to-no enhancement (Figure 1) [6,7,8,9]. Their imaging appearance will mirror that of fat and appear as homogeneous and isoechoic or hyperechoic on US; strongly hypodense on CT; and with the same MR signal intensity of subcutaneous and intrabdominal fatty tissues. Of note, Dual Echo sequences will not show signal dropout since lipomas contain extracellular fat.

When inflammation, necrosis, or infarction occur, a heterogeneous appearance [9] with calcifications, thin septa (<2 mm), or bands of muscle fibers can be appreciated [7,8]. In these cases, differentiation with liposarcoma cannot be made at imaging, and biopsy or surgical resection is recommended [6], especially in bigger lesions. When solid components are present, liposarcoma should always be ruled out [6,9].

Lipomas are commonly painless, have no malignant potential, and do not require follow-up. However, subcutaneous lipomas could be surgically removed for symptomatic relief, pathologic confirmation, cosmetic reasons, or if there is an increase in size [9,10]. In these cases, pre-operative CT can be performed to depict their extent. Complete surgical excision including the capsule is advocated to avoid local recurrence. In painful subcutaneous lipomas, either isolated or syndromic (e.g., Dercum’s Disease), management includes options such as liposuction or lidocaine applications [10].

### 2.2. Desmoid Tumors

Desmoid tumors (i.e., “aggressive fibromatosis”) [11] are benign myofibroblast connective tissue tumors, usually occurring in women between 25 and 40 years old [12,13]. Desmoid tumors can be either intra- or extra-abdominal [11], typically do not cross the midline and do not metastasize, but may show infiltrative growth and have a high tendency to recur after resection [7,11,13]. Differential diagnosis includes sarcomas, metastasis, injection granulomas, postsurgical fibrosis, and endometriosis [12].

**Figure 2:**
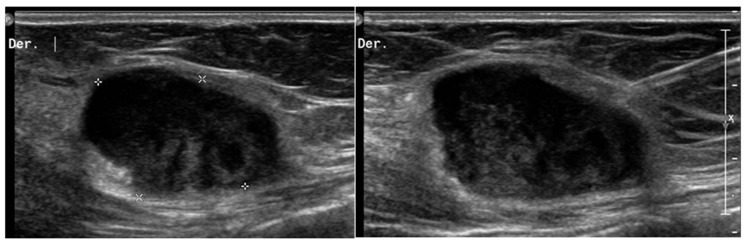
Desmoid tumors on US. A 45-year-old woman with desmoid tumor of the abdominal wall. B-mode US scan shows a rounded, poorly circumscribed mass in the context of the abdominal muscles (*), heterogeneously hypoechoic, but similar to muscles that underwent biopsy (right picture).

On imaging, desmoid tumors resemble the surrounding muscles [11,12,13]. Hypoechoic, poorly circumscribed, with internal vascular flow on US (Figure 2) [7,13], they are commonly briskly hyperattenuating and homogeneous on contrast-enhanced CT, with either circumscribed or ill-defined margins and without washout [13].

**Figure 3:**
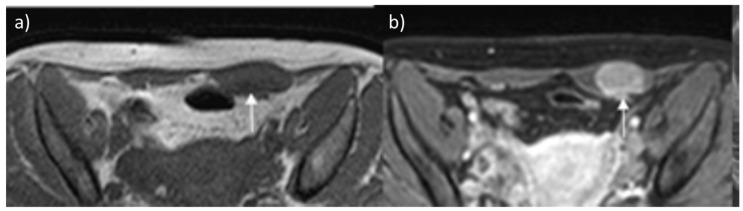
Desmoid tumor on MR. Axial T2 (**a**) and T1-weighted (**b**) contrast-enhanced scan show the typical appearance of a long-standing desmoid tumor (arrow), which is almost indistinguishable from surrounding muscles. After contrast, the mass shows brisk and homogeneous enhancement.

Larger tumors may appear heterogeneous, as a result of bleeding or necrosis, and may contain calcifications [11,12,13]. On MR, desmoid tumors have low-to-intermediate T1-weighted (T1-w) signal and high T2-weighted (T2-w) foci, which during subsequent follow-ups become isointense to the surrounding muscles (Figure 3) [3,4,12]. Imaging appearance overlaps with other abdominal wall masses, so tissue sampling is required when desmoids are suspected [1].

The first sign of a desmoid tumor may be a firm, painless lump. When they develop in flexible, elastic tissues or deep spaces, the tumor can often push normal tissue out of its way as it grows, causing vague symptoms. In the past years, direct complete surgery was the standard primary treatment modality; however, in recent years a paradigm shift towards a conservative management has been introduced in order to prevent local recurrences, as they are usually more aggressive than the original tumor [14]. Moreover, recent studies showed no difference in event-free survival and long-term disease control between patients undergoing surgery and those managed with a conservative approach among patients presenting with favorable locations, such as the abdominal wall [14,15].

### 2.3. Hemangioma

**Figure 4:**
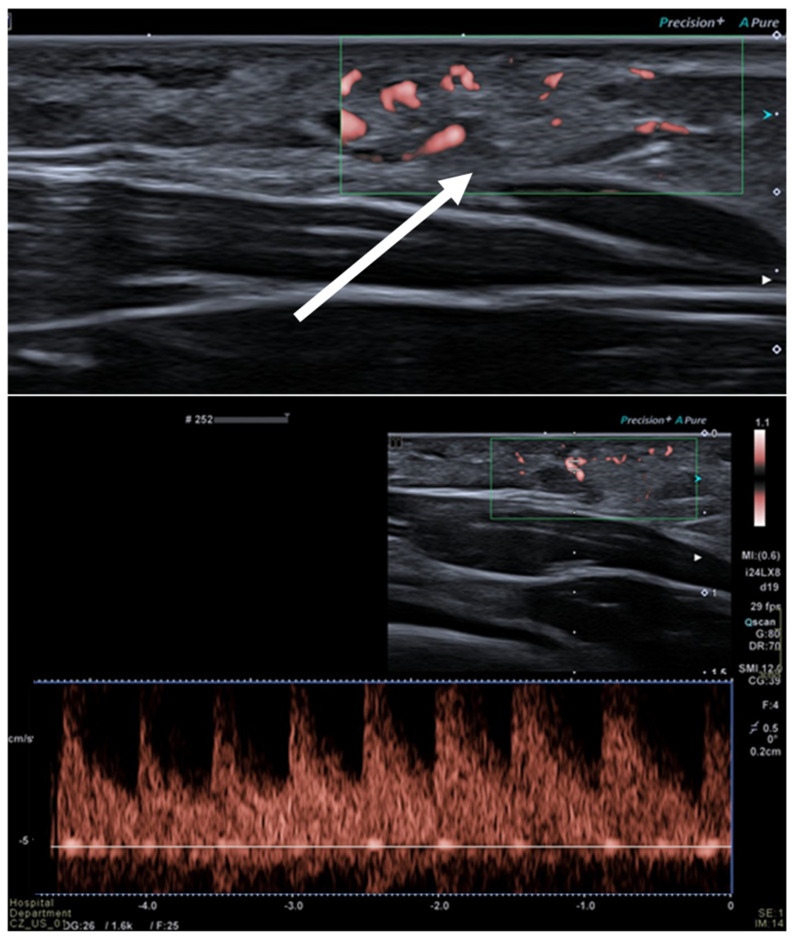
Intra-muscular hemangioma in a 2-year-old male patient. US with a linear transducer shows the presence of an echogenic, well-defined mass along the surface of the anterior abdominal wall (arrow, **upper** picture). This lesion shows prominent vascularity, with high-flow arterial waveforms on Doppler integration (**bottom** picture), hallmarks of infantile hemangiomas.

Abdominal wall hemangiomas are rare mesenchymal tumors (<1%), mainly occurring in children [16,17]. US will demonstrate iso- or hypoechoic lesions, similar to surrounding muscles, with ill-defined borders, in the context of abdominal wall muscles [16,18], presenting feeding vessels with arterial parenchymal waveforms on color Doppler, usually with high resistive index (Figure 4) [16,17,18].

On CT, hemangiomas appear as lesions without clearly infiltrating characteristics, usually with phleboliths, which are punctuate calcifications, suggestive for the diagnosis [8]. In post-contrast sequences, hemangiomas show early homogeneous enhancement or progressive centripetal enhancement [18].

On MR, abdominal wall hemangiomas are isointense or only slightly hyperintense on T1-w images, and hyperintense on T2-w ones [3,8,17], with low-signal spots corresponding to phleboliths, that will create the so-called “blooming artifact” on Dual Echo sequences [16]. Two clues for the diagnosis are the presence of a fat-rim in intramuscular hemangiomas (i.e., an outer loss of signal in all fat-suppressed sequences) [4] and the presence of fluid–fluid levels within the lesion, for more than two thirds of its diameter [4,19], related to slow-moving flows within the hemangioma [19].

Although true hemangiomas have no malignant potential, all management strategies should include regular follow-ups.

Conservative management is the first line of treatment for nearly all isolated intramuscular hemangiomas. When the degree of pain or functional impairment warrants more intervention, nonoperative measures may decrease symptoms, and, if nonresponsive, options include conservative management, topical or systemic corticosteroids, embolization, sclerotherapy, and surgical excision [20].

### 2.4. Nerve Sheath Tumors

**Figure 5:**
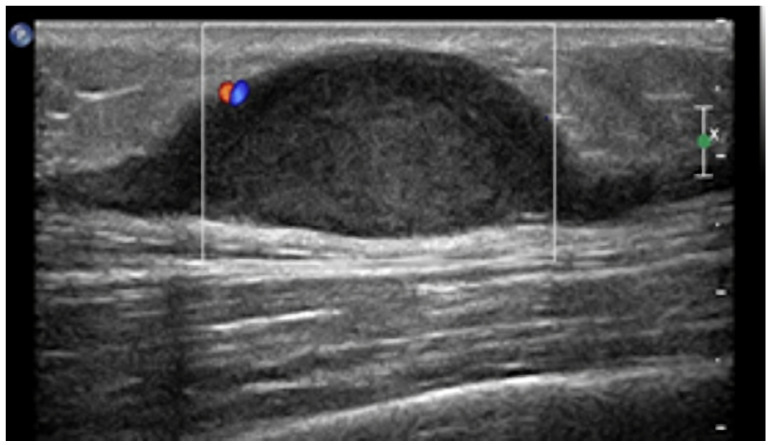
US appearance of schwannomas. Most schwannomas arise from the nerve sheath of peripheral nerves and occur below or at the level of the subcutaneous fat layer and, as shown, appear as hypoechoic fusiform mass with small blood flow on color Doppler and, sometimes, peripheral nerve continuity.

Benign nerve sheath tumors (NSTs) are mainly comprised of neurinomas and schwannomas and are typically located along the course of major peripheral nerves [1,7,8,13]. If multiple lesions are seen, neurofibromatosis type I (NF1) should always be ruled out, [1,3,8,13] as it is associated with a higher risk of malignant degeneration of NSTs and therefore requires periodical follow-up [3]. On US, NSTs appear as smooth well-defined lobulated masses (Figure 5), homogenously hypoechoic on US, without intralesional vascularization, hypoattenuating on CT, with minimal-to-no enhancement.

**Figure 6:**
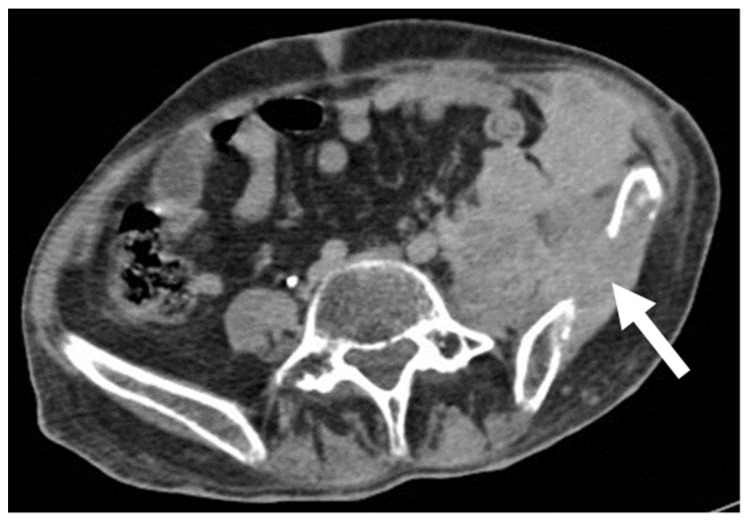
Malignant schwannoma in a 37-year-old man with neurofibromatosis type 1. Portal venous, axial, contrast-enhanced CT scan demonstrates an infiltrative disease, invading the left iliac bone (arrow), and with heterogeneous contrast enhancement.

Neurofibromas and schwannomas contain a solid and a myxoid part and will show low signal on T1-w images, with heterogeneous appearance on T2-w images. A “target sign” could also be appreciated on T2-w sequences [1,2,3,4,8], consisting of a peripheral high intensity area, with central low signal, reflecting myxoid degeneration [1,13]. After gadolinium, a “reverse target sign” will appear, given that the enhancement is only central [21]. Another diagnostic tip is the presence of the “split fat sign”, a rim of fat around the tumor [8]. If malignant degeneration occurs, inhomogeneous enhancement and rapid growth will appear, together with ill-defined margins, infiltration, and destructive changes in adjacent bone structures (Figure 6). NSTs of the abdominal wall are usually asymptomatic or just slightly symptomatic and can be managed through follow-up, but are exclusively treated by excision. Prognosis is good with low recurrence rates [22].

## 3. Imaging Findings: Malignant Primary Neoplasms

Table 2 summarizes main imaging features of malignant abdominal wall neoplasms.

### 3.1. Sarcomas

Sarcomas are rare mesenchymal tumors, often large, with poorly defined margins, which can arise in the subcutaneous tissue or in the musculature of the abdominal wall [2,3,23]. The most common types are liposarcomas and leiomyosarcomas [3].

**Figure 7:**
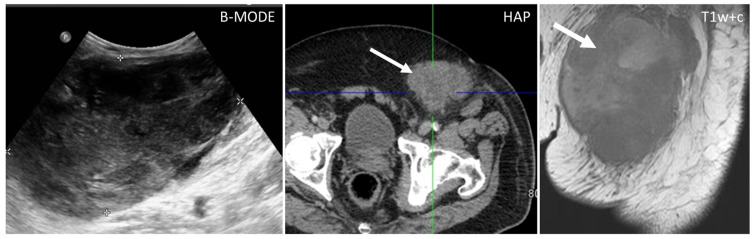
US, CT, and MR appearance of sarcomas of the abdominal wall in a 69-year-old male with a palpable mass. B-mode US (first picture) shows the presence of a large, heterogeneously hypoechoic mass, and anechoic areas corresponding to necrosis. Axial, contrast-enhanced CT scan on arterial phase (second picture) shows the presence of a poorly defined mass (arrow) with hazy borders and patchy enhancement. Sagittal, contrast-enhanced MR T1w image (third picture) shows the typical early diffuse enhancement of sarcomas.

Sarcomas of the abdominal wall are usually non-vascularized solid masses, with necrotic or fluid areas [23], giving the lesion a heterogeneous aspect, mostly hypoechoic on US, with anechoic parts corresponding to fluid degeneration. CT will better characterize extension, staging, and the anatomic origin and will demonstrate hyperattenuating, soft-density structures, with early heterogeneous enhancement (Figure 7) [2,24].

**Figure 8:**
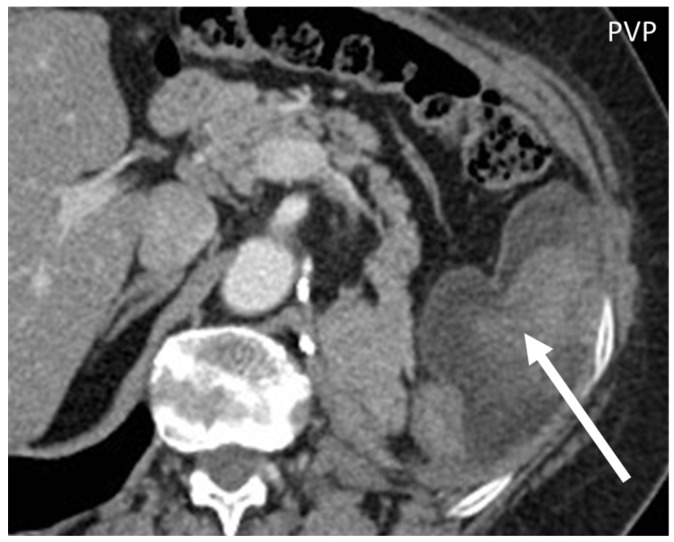
Axial, portal venous phase CT shows the appearance of a well-differentiated liposarcoma (arrow) of the left transverse muscle in a 63-year-old woman, presenting as a lobulated mass with fat peripheral attenuation and slight enhancement.

**Figure 9:**
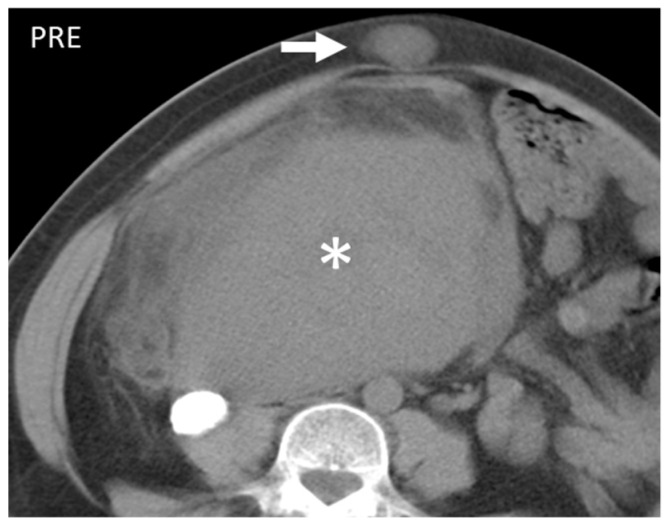
Axial unenhanced CT scan shows a large mass (*) and a lesion on the subcutaneous fat of the abdominal wall. Histological examination revealed it to be a leiomyosarcoma and had a localization on the abdominal wall (arrow). This entity, when superficial, presents as well-demarked and homogeneous and, contrary to liposarcomas, does not show fatty component.

**Figure 10:**
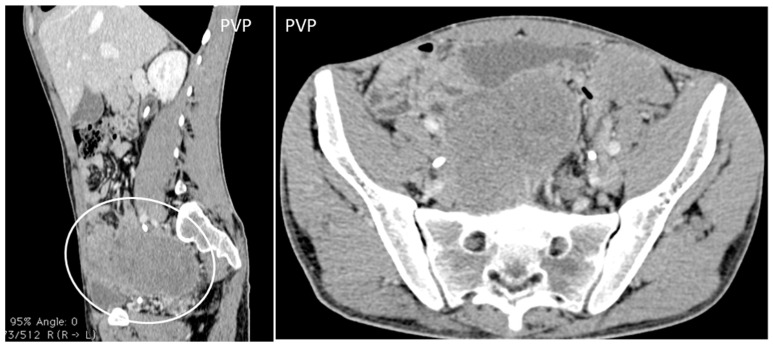
A 26-year-old patient coming to medical attention for abdominal tenderness and sudden weight loss. Contrast enhanced sagittal and axial CT scan shows the characteristic CT findings of desmoplastic small round cell tumor (DSRCT): bulky multiple, soft-tissue masses with modest enhancement, and omental, serosal, and rectovesical involvement with rare calcifications, invading the anterior abdominal wall.

Among sarcomas we can distinguish:

- **Liposarcoma**: well-defined fat and myxoid areas will be seen inside a heterogeneously enhancing lesion, with a characteristic sharp demarcation among fatty and nonfatty elements. Calcifications are rare (Figure 8) [3,4,5,6,7,8,9,23].
- **Leiomyosarcomas**: seen on the abdominal wall as either a primary process or as an extension of an intra-abdominal process [23,24]. Leiomyosarcomas demonstrate heterogeneous attenuation and signal intensity, with irregular peripheral enhancement and enhancing solid portions, mixed with hemorrhagic and necrotic areas [24]. Fatty components are absent (Figure 9) [23,24].
- **Gastrointestinal Stromal Tumors (GIST):** either primary (extraintestinal GIST, “EGIST”) [25,26] or secondary GIST of the abdominal wall are rare. When extended (>5 cm), GIST may have an aggressive behavior [25]. CT is the imaging modality of choice, showing heterogeneous vivid enhancement, and variable amount of necrosis. Peculiar findings include calcifications and cystic degeneration [27,28,29].
- **Desmoplastic Small Round Cell Tumor (DSRCT):** rare, highly aggressive sarcoma of adolescents, which primarily involves the serosal surfaces of the abdominal cavity infiltrating the abdominal wall [30,31,32]. Classic findings include bulky multiple, hypoattenuating, soft-tissue masses, with omental, serosal, and rectovesical involvement [31,32] and typical punctate or amorphous calcifications [32]. Modest heterogeneous enhancement is seen on arterial phase, without prolonged enhancement or portal washout [31,32] (Figure 10). On MR, DSRCTs are heterogeneously iso- to-hypointense on T1-w images, with hyperintense foci due to intratumoral hemorrhage [32].

### 3.2. Subcutaneous Lymphoma

**Figure 11:**
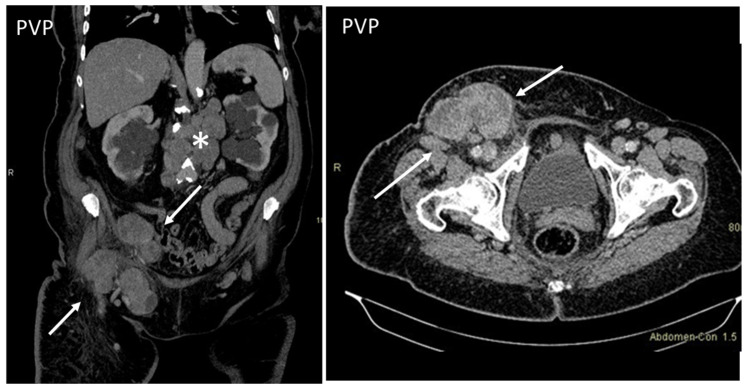
Axial, contrast-enhanced CT on portal venous phase shows enlarged inguinal (arrows) and retroperitoneal (*) lymph nodes in a patient with histologically proven non-Hodgkin’s lymphoma.

Lymphoma may involve the subcutaneous tissue, muscles, and skin of the abdominal wall, as a primary process, by contiguous extension or by hematogenous and lymphatic spread [11,12,13]. The diagnosis is primarily based on diffuse or systemic lymphatic involvement and hepatosplenomegaly [33]. Findings include large masses, nodal or confluent nodal structures, small nodules (<1 cm), disseminated myositis, or panniculitis [13]. On US, subcutaneous lymphoma presents as a well-defined, hypoechoic lesion without increased vascularity [34]. On cross-sectional imaging, confluent masses with homogeneous or heterogeneous enhancement can be appreciated (Figure 11) [13].

On MRI, abdominal wall lymphomas show high T2 signal, rarely low-to-intermediate [34], homogenous signal, lack of central necrosis in large lesions [34], lymphangitis, and the “wrapped-around” sign of lymphoma around bony structures [34].

### 3.3. Metastasis

**Figure 12:**
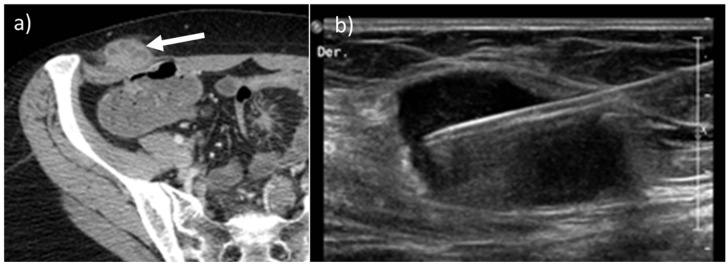
Axial, contrast-enhanced CT (**a**, arrow) on portal venous phase of a 45-year-old female with history of ovarian cancer treated with laparoscopic surgery demonstrates the presence of a solitary metastatic mass along the right abdominal subcutaneous fat. After laparoscopic surgery, implant tumors can be found along the course of the trocars. Nonetheless, a biopsy was made, as shown on US B-mode picture (**b**),that confirmed the presence of disease.

Around 5–10% of all oncologic patients may develop abdominal wall metastases (AWMs) [11]. Secondary AWMs derive from hematogenous or lymphatic spread, direct invasion, or needle-tract seeding [1,13]. When the abdominal wall is the sole potential site of metastasis seen on imaging, biopsy is still recommended (Figure 12) [1].

Imaging findings are nonspecific [1,2,3] but include single or multiple soft-tissue nodules that resemble the primary lesion in both appearance and contrast behavior [1]. On CT and MR, heterogeneous or peripheral enhancement will usually be seen [13,35], an appearance called “pseudo-target sign” [4].

**Figure 13:**
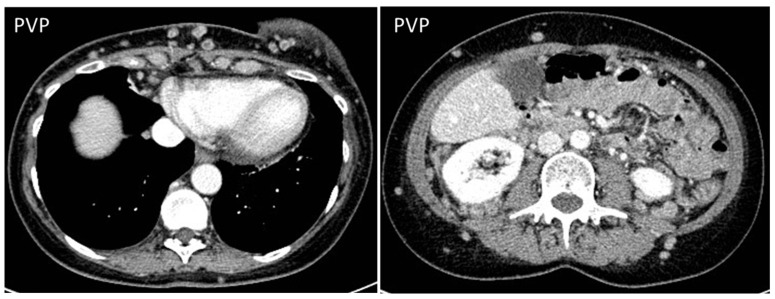
CT on portal venous phase in a woman who recently underwent quadrantectomy for breast cancer shows multiple nodules spreading from the site of the operation to the subcutaneous fat of the anterior and posterior abdominal wall.

Breast cancer is one of the most common causes of abdominal wall and subcutaneous metastasis, with an incidence between 5 and 24% (Figure 13) [36].

**Figure 14:**
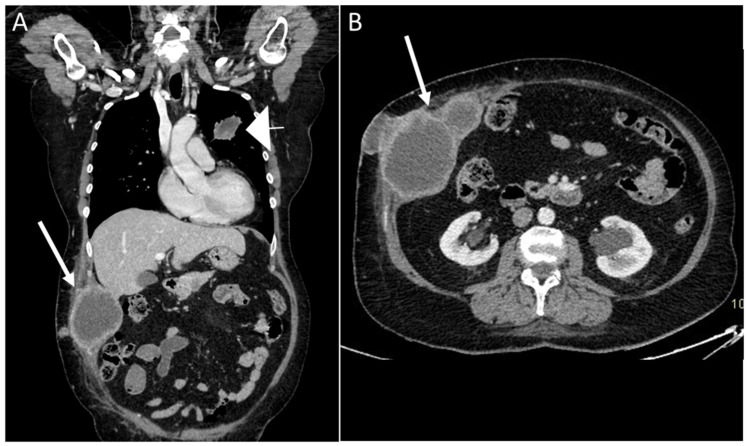
A 68-year-old woman with a palpable solid mass on the abdominal wall. Coronal and axial CT scan on portal venous phase showed the presence of a primary lung cancer tumor (**A**), arrowhead) with multiple implants, including the abdominal wall (arrows, **A** and **B**).

Melanoma metastasis are also common, and have a tendency to bleed [11,35,36,37,38,39,40]. They are hypervascular in 10–17% of cases, with high T1 signal on MR, due to the presence of melanin. Other primary tumors that may lead to AWMs include lung cancer [39], gynecological malignancies, and colorectal cancer (Figure 14) [40,41].

AWMs can also be a consequence of laparoscopy, needle biopsies, and radiofrequency ablation [37] in multiple cancers, including hepatobiliary malignancies, neuroendocrine tumors, peritoneal carcinomatosis, and gastric cancer, reaching a prevalence of 16–47% in gynecologic malignancies [35].

**Figure 15:**
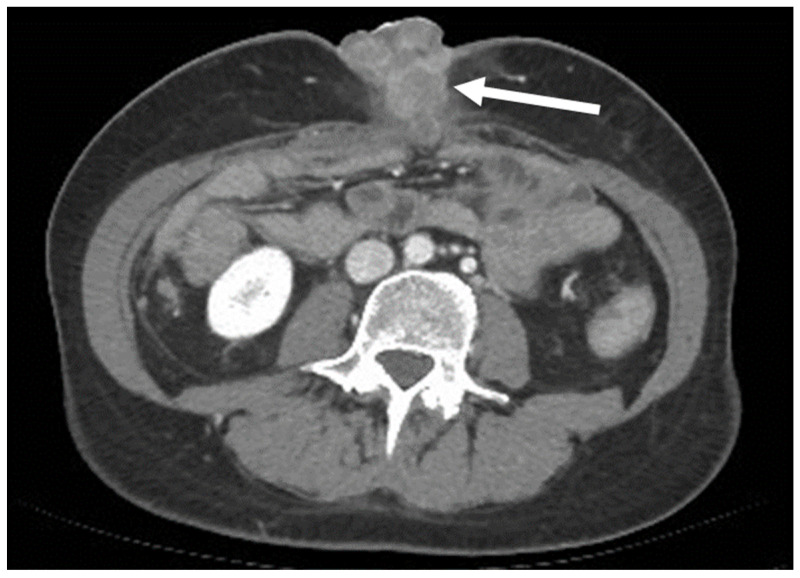
A 50-year-old woman with an umbilical mass. Axial, portal venous phase CT scan shows a collection of masses with heterogeneous, peripheral enhancement metastases along the navel (arrow). It was then demonstrated that the primary site of the tumor was an undifferentiated pancreatic carcinoma.

A particular AWM appearance is “Sister Mary Joseph’s Nodule” (SMJN), a metastatic deposit located in the umbilicus. SMJN can be the first complaint of an unknown cancer and it is indicative of an aggressive disease [42,43,44,45,46]. The most common primary sites of SMJN are, in order of frequency, pancreato-biliary, gastrointestinal, colorectal, ovarian, and endometrial adenocarcinomas, [35,38,43,44,45,46] even though one fourth of cases will derive from undifferentiated cancers (Figure 15) [46]. SMJN can be the first known sign of a malignancy, as the appearance of the navel will alert the patient to seek medical attention.

## 4. Conclusions

The spectrum of abdominal wall neoplasms is wide, ranging from benign tumors that can be conservatively managed to malignant ones, which require treatment.

The role of the radiologist is fundamental in curtailing the differential diagnosis and correctly interpreting these processes, especially in the case of incidental findings.

Some imaging clues may help to narrow the differential diagnosis, indicating the need for further diagnostic work-up in case of larger lesion size, edema, neurovascular involvement, or peripheral or inhomogeneous dynamic enhancement. Some benign tumors may have atypical features or may lead to complications, and this may lead to diagnostic challenges at imaging and indicate the need for a biopsy to rule out malignancy.

## Figures and Tables

**Table 1:** Key imaging features of benign neoplasms of abdominal wall. List of abbreviations: T2-weighted images = T2w; T2-weighted images = T1w; contrast = c; diffusion weighted imaging = DWI; apparent diffusion coefficient map = ADC.

Imaging Findings—Benign Neoplasms			
Lesion	Ultrasounds (US)	Computed Tomography (CT)	Magnetic Resonance (MRI)
Lipoma	Homogeneous and isoechoic or hyperechoic in most cases Hypoechoic in 20% of cases. No internal vascular flow	Strongly hypoattenuating No contrast enhancement	T2w: moderately hyperintense in non-fat suppressed images T1w+c: no contrast enhancement DWI: no restriction Dual-Echo: no signal drop on out-of-phase
Desmoid Tumors	Hypoechoic Poorly circumscribed Internal vascular flow	Circumscribed or ill-defined margins Commonly moderate to high, homogenous enhancement. Tendency to fade, no washout Larger tumors: calcifications, bleeding, or necrosis	T1w: low-to-intermediate signal. High if bleeding occurred. +c: commonly enhancing T2w: low signal, with high signal foci DWI: no significant restriction ADC map: higher values than malignancies
Hemangioma	Iso-to-hypoechoic Internal vascular flow Doppler: arterial waveforms	+/− Phleboliths No infiltrating characteristics Early homogeneous enhancement or progressive centripetal enhancement	T1w: slightly high T2w: high “Blooming artifact” (phleboliths) Fluid-fluid levels (>2/3) +c: enhancement
Nerve Sheath Tumors	Usually hypoechoic No internal vascular flow	Low to intermediate attenuation Contrast enhancement, usually homogeneous	T1w: low T2w: heterogeneously high Cystic degeneration in larger tumors “Target” and “reverse target sign” “Split fat sign”

**Table 2:** Key imaging features of malignant neoplasms of abdominal wall. List of abbreviations: T2-weighted images = T2w; T2-weighted images = T1w; contrast = c; diffusion weighted imaging = DWI; apparent diffusion coefficient map = ADC; gastrointestinal stromal tumor = GIST; desmoplastic small round cell tumor = DSRCT.

Imaging Findings—Malignant Neoplasms			
Lesion	Ultrasounds (US)	Computed Tomography (CT)	Magnetic Resonance (MRI)
Malignant Schwannoma	Heterogeneously hypoechoic	Large/growing masses Irregular margins Heterogeneous enhancement Infiltrating surrounding bones	T1w: heterogeneous T2w: low DWI restriction
Sarcomas			
Liposarcomas	Heterogeneous with lipomatous parts, solid parts, calcifications, cystic degeneration (necrosis)	Sharp demarcation among fatty and nonfatty elements Heterogeneous enhancement +/− calcifications Ill-defined margins, invasion of nearby structures	Low-grade lesions: fat signal with septa/ enhancement/small solid foci High-grade lesions: heterogeneous signal depending on the prevalence of each component DWI restriction
Leiomyosarcomas	Heterogeneous	Solid, ill-defined mass with necrotic changes Slight, heterogeneous enhancement	T1w: isointense to muscles T2: high DWI restriction
GIST	Heterogeneous +/− necrosis and cystic degeneration	Soft tissue heterogeneous mass Heterogeneous, enhancement +/− necrosis and cystic degeneration	T1w: low (high if necrosis) T2w: high +/− necrosis and cystic degeneration
DSRCT		Bulky multiple, hypoattenuating, soft-tissue masses Amorphous calcifications Modest heterogeneous enhancement without washout	T1w: heterogeneously iso-to-hypointense; hyperintense foci if hemorrhage T2w: heterogeneously slightly hyperintense
Subcutaneous Lymphoma	Multiple, well-defined hypoechoic	Confluent masses with homogeneous/heterogeneous slight enhancement, without washout Nodal involvement	T1w: iso-to-hypointense masses T2w: iso-to-hyperintense DWI: restriction
Metastasis	Mirroring imaging characteristics of primary tumor: usually solid masses with target appearance and rim vascularization, with DWI restriction on MRI.		

## Data Availability

Not applicable.
